# A survey of primary-care pediatricians regarding the management of *Helicobacter pylori* infection and celiac disease

**DOI:** 10.1186/s13584-019-0357-x

**Published:** 2019-12-27

**Authors:** Amir Ben Tov, Wasef Na’amnih, Amna Bdair-Amsha, Shlomi Cohen, Judith Tzamir, Gabriel Chodick, Khitam Muhsen

**Affiliations:** 1 Maccabi Research Institute, Maccabi Healthcare Services, Tel Aviv-Yafo, Israel; 2 Pediatric Gastroenterology Unit, “Dana-Dwek” Children’s Hospital, Tel Aviv Sourasky Medical Center, Tel Aviv-Yafo, Israel; 3 Department of Epidemiology and Preventive Medicine, School of Public Health, Sackler Faculty of Medicine, Tel Aviv University, 6997801 Tel Aviv, Israel

**Keywords:** Survey, *Helicobacter pylori*, Celiac disease, Diagnosis, Treatment, Guidelines, Pediatricians

## Abstract

**Background:**

Adherence of primary-care pediatricians to guidelines in pediatric gastroenterology is essential to achieve optimal clinical outcomes. The study aim was to examine adherence of primary-care pediatricians to the European and North American Societies for Pediatric Gastroenterology, Hepatology and Nutrition guidelines on the management of *Helicobacter pylori* (*H. pylori*) infection and celiac disease.

**Methods:**

We conducted a cross-sectional study during March–July 2017 using the survey platform of Maccabi Healthcare Services, the second largest state-mandated health organization in Israel. We sent the study questionnaire to a random sample of 300 pediatricians via electronic mails and to increase the response rate, we performed a telephone interview. Overall, 108 (36%) pediatricians provided completed questionnaires.

**Results:**

Using professional guidelines for the management of *H. pylori* infection and celiac disease was reported by 34 and 37% of pediatricians, respectively. Referral to *H. pylori* testing was reported by 78 and 52% of pediatricians in children with suspected duodenal ulcer and unexplained iron deficiency anemia, respectively, with the stool antigen enzyme immunoassay being mostly (51%) used as the first choice diagnostic test. Most pediatricians reported prescription of triple therapy; proton pump inhibitors/clarithromycin/amoxicillin (59%) or metronidazole (21%). For celiac disease, overall adherence to all guidelines was high both for initial evaluation and for confirmation of diagnosis.

**Conclusions:**

Adherence to the guidelines on management of *H. pylori* infection was low, while adherence to the guidelines on celiac disease management was high among primary-care pediatricians. Educational interventions are needed to improve *H. pylori* infection management among primary-care pediatricians.

## Background

Gastrointestinal complaints such as abdominal pain, nausea, and diarrhea are common in the pediatric practice [1]. Clinical guidelines based on synthesis of evidence by experts in the field of pediatric gastroenterology and nutrition provide high-quality summary of recommendations on testing, treatment and follow-up of pediatric patients with various gastrointestinal conditions such as *Helicobacter pylori (H. pylori)* infection [2, 3], celiac disease [4], and other conditions. These guidelines aimed to create a standard of care based on best available evidence with emphasis on the diagnostic process in each condition.

According to the European Society for Pediatric Gastroenterology Hepatology and Nutrition and North American Society for Pediatric Gastroenterology, Hepatology and Nutrition (ESPGHAN and NASPGHAN) guidelines, esophagogastroduodenoscopy is recommended as a first choice for the diagnosis of *H. pylori* infection. The recommended first-line eradication regimens included triple therapy with a PPI/amoxicillin/clarithromycin or an imidazole or bismuth saltsamoxicillinan imidazole or sequential therapy. Confirmation of *H. pylori* eradication using non-invasive reliable tests such as the urea breath test (UBT) and stool antigen detection enzyme immunoassays (EIA) should be done 4–8 weeks after completing therapy. According to the 2012 ESPGHAN guidelines [4], the serological assays constitute the first step in the diagnosis of celiac disease. Patients testing positive for specific tissue transglutaminase type 2 (TG2) antibody should be referred to a pediatric gastroenterologist for further diagnostic workup that might include anti-endomysium antibodies and biopsy, depending the serology results.

Primary-care pediatricians usually are the first to be contacted by parents regarding their child’s illness. These physicians make most of the decisions regarding referral to diagnostic tests and treatment of children with gastrointestinal illnesses. Primary-care pediatricians vary according to their education, sub-specialty and experience. Adherence to guidelines created by professional societies regarding the diagnosis and treatment of children with gastrointestinal illnesses is expected to ensure safe and optimal treatment and achieve satisfactory clinical endpoints. However, utilization and adherence of these guidelines by primary-care pediatricians remains unclear. The aim of the current study was to examine adherence of primary-care pediatricians to the ESPGHAN/NASPGHAN guidelines for the diagnosis and treatment *H. pylori* infection [2, 3] and the ESPGHAN guidelines on celiac disease [4], as models for infectious and non-infectious chronic gastrointestinal illnesses, respectively.

## Methods

### Study design and population

We conducted a cross-sectional study during March–July 2017 using the survey platform of Maccabi Healthcare Services (MHS), the second largest state-mandated health organization in Israel. A random sample of 300 primary care pediatricians was selected among all pediatrician’s employees of MHS. Overall, 113 pediatricians agreed to participate in the study, of those 73 were successfully contacted by the email messages and 40 by telephone, while five pediatricians did not complete the survey, thus leaving 108 (36%) participants in the analysis.

### The instrument

The study team constructed a questionnaire (Additional file 1). For some Likert scale survey’s questions, we constructed a dichotomous variable by combining the categories (always and usually-yes) into one category and the other categories (usually-no and never) to the second category. The questionnaire consisted of questions on the utilization of professional guidelines in the diagnosis and treatment of *H. pylori* infection and celiac disease and the physicians’ referral patterns to diagnostic tests and treatment of these conditions. We used The 2011 ESPGHAN/NASPGHAN guidelines on *H. pylori* infection [2] and the 2012 ESPGHAN guidelines of celiac disease [4] as the reference in our study. Information on characteristics of all selected pediatricians was obtained from the MHS database on the physician’s age in years, sex, the year in which he/she began to work at MHS and type of employment/contract with MHS [being a contractor vs. employee of MHS). Information on number of years since board certification of the participating physicians was obtained through the questionnaire.

We sent the questionnaire to the physicians in the study sample via the electronic mail system of MHS. To increase the response, we sent two messages in different occasions 3–4 weeks apart. The study team contacted physicians who did not open the survey link by phone; and interviewed those who were successfully reached and agreed to participate in the survey.

### Statistical analysis

We examined differences between responders and non-responders in background characteristics using the chi-square test or Fisher’s exact test for categorical variables and Student’s *t* test for continuous variables. Categorical variables were described using frequencies and percentages and continuous variables were described using means and standard deviation (SD). Results from unweighted and weighted analyses are presented. The weights were determined using the inverse probability weighting method [5]. The probability to participate in the study was obtained from multiple logistic regression model in which the dependent variable was participating on the study (yes or no, coded 1 and 0, respectively) and the independent variables were age, sex and the year of starting to work at MHS. The inverse of this probability was used as the weight. *P* < 0.05 was considered statically significant. We analyzed the data using SPSS version 25 (IBM, New York, United States).

## Results

There were no significant differences in age, sex, years since board certification, employment contract and the year of beginning working at MHS between responders and non-responders (Table 1).

**Table 1:** Comparison between the respondents and non-respondents

Variable	Respondents *N* = 104*	Non-respondents *N* = 196	*P* value
Mean age (SD), years	55.1 (10.7)	57.2 (9.8)	0.09
Sex, males	58 (56%)	108 (55%)	0.9
Seniority (Employed at MHS for more than 7 years)	27 (26%)	42 (21%)	0.4
Employment time, independent (contractor) physician	99 (95%)	178 (91%)	0.3

*Missing data: Four respondents

*MHS* Maccabi Healthcare Services, *SD* Standard deviation

### *H. pylori* infection

Among 103 participants who replied to the question regarding using guidelines, 35 (34%) reported utilization of any guidelines for the diagnosis of *H. pylori* infection.

Testing for *H. pylori* infection in patients with suspected duodenal ulcer was reported by 78% of the participants compared to 52 and 47% in patients with unexplained/ refractory iron deficiency anemia (IDA) and first-degree relatives of gastric cancer patients, respectively. All of these conditions should promote testing for *H. pylori* according to the guidelines. However 44% reported testing of children with recurrent abdominal pain to the diagnosis of *H. pylori* infection, where there is a recommendation not to test. Nearly half of the participants reported testing for stool antigen detection EIA as their first-choice diagnostic test, followed by the UBT (27%).

Most (59%) participants reported that they would prescribe triple therapy with proton pump inhibitors (PPIs)/clarithromycin/amoxicillin as first-line treatment, 21% reported prescription of triple therapy, but using metronidazole instead of clarithromycin. Forty percent reported prescription of anti-*H. pylori* therapy for 10 days, and 34% for 14 days. More than half reported that they do not refer their patients to follow-up examination after *H. pylori* treatment if symptoms resolved. In case of treatment failure, most participants (71%) reported that they would refer their patient to a specialist in gastroenterology. The weighted analysis yielded similar results (Table 2).

**Table 2:** Self-reported practices of primary-care pediatricians regarding the management of *H. pylori* infection in children

	Number/ Total (percent)	Weighted percent^*^	Relevant Recommendations [5]
*Reasons for testing for H. pylori diagnosis in the case of* ^**^			
Suspected duodenal ulcer	76/98 (78%)	78%	Recommended
First-degree relatives of gastric cancer patients	45/96 (47%)	46%	Testing for *H. pylori* may be considered
Recurrent abdominal pain	45/101 (44%)	46%	Not recommended
Unexplained IDA	51/99 (52%)	52%	Recommended in children with refractory IDA, in which other causes have been ruled out
*First choice diagnostic test for H. pylori*			
UBT	27/102 (27%)	25%	
Gastroscopy	2/102 (2%)	2%	The initial diagnosis of *H. pylori* infection should be based on either a positive histopathology plus a positive rapid urease test or a positive culture.
Specialist in gastroenterology	20/102 (20%)	19%	
Stool antigen EIA	52/102 (51%)	53%	
Serology	1/102 (1%)	1%	
*Prescription of first line therapy*			Triple therapy with a PPI/ amoxicillin/ clarithromycin or an imidazole or bismuth saltsamoxicillinan imidazole or sequential therapy. Antibiotic susceptibility testing for clarithromycin is recommended before in areas with a high resistance rate (> 20%).
PPIs/clarithromycin/ amoxicillin	60/102 (59%)	58%	
PPIs/ amoxicillin / metronidazole	21/102 (21%)	22%	
PPIs/clarithromycin/ amoxicillin /metronidazole	4/102 (4%)	4%	
Refer to a specialist in gastroenterology	16/102 (16%)	16%	
*Duration of treatment*			7 to 14 days
7 days	15/102 (15%)	14%	
10 days	41/102 (40%)	42%	
14 days	35/102 (34%)	34%	
Refer to a specialist in gastroenterology	11/102 (11%)	11%	
*Follow-up*			A reliable noninvasive test to confirm eradication at least 4–8 weeks following completion of therapy (UBT or stool EIA).
UBT at least 1 month after therapy	19/102 (19%)	19%	
Refer to a specialist in gastroenterology	11/102 (11%)	11%	
Stool antigen detection EIA at least 1 month after therapy	17/102 (17%)	17%	
Do not refer to follow-up test if symptoms resolved	55/102 (54%)	53%	
*In case of treatment failure*			EGD, with culture and susceptibility testing including alternative antibiotics; modification of therapy.
Refer to a specialist in gastroenterology	72/102 (71%)	71%	
Do nothing if symptoms resolved	14/102 (14%)	13%	
The same treatment for longer duration	4/102 (4%)	5%	
Recommend a different treatment	12/102 (12%)	12%	

^*^Inverse probability weighting; ^**^Physicians who answered “always” or “usually”. *EIA* Enzyme immunoassay, *EGD* Esophagogastroduodenoscopy, *IDA* Iron deficiency anemia, *IgA* Immunoglobulin A, *IgG* Immunoglobulin G, *PPIs* Proton pump inhibitors, *UBT* Urea breath test

### Celiac disease

Forty (37%) participants reported using professional guidelines for the diagnosis and treatment of celiac disease.

Most participants (93%) reported that they suspect their patient to have celiac disease if he/she had chronic/intermittent diarrhea, growth impairment (97%), IDA (94%) or complaints of abdominal pain (85%). The majority of the participants reported recommending screening for celiac disease for patients with autoimmune disease and first-degree relatives of celiac disease patients: 92 and 98%, respectively. All participants reported recommending their patient gluten-free diet only after final diagnosis of celiac disease, 98% reported recommending their celiac disease patients an annual follow-up to monitor physical growth and disease complications, while 84% reported recommending a follow-up by a specialist in gastroenterology (Table 3). The weighted analysis yielded similar results (Table 3).

**Table 3:** Self-reported practices of primary-care pediatricians on the management of celiac disease (CD) in children

	Number/ Total (percent)	Weighted percent*	Comment [6]
*Reasons for testing for CD*			Patients with CD may present with a wide range of symptoms and signs or be asymptomatic
Chronic/intermittent diarrhea	100/108 (93%)	92%	
Growth impairment	105/108 (97%)	98%	
IDA	102/108 (94%)	95%	
Abdominal pain	92/108 (85%)	86%	
*Reasons for screening for CD*			First-degree relatives with CD, type 1 diabetes, Down syndrome, Turner syndrome autoimmune thyroid disease, Williams syndrome, IgA deficiency and autoimmune liver disease.
Autoimmune diseases, e.g., type 1 diabetes	99/108 (92%)	91%	
Down syndrome	66/108 (61%)	62%	
First-degree relatives of CD patients	106/108 (98%)	98%	
Referral for diagnosis in suspected cases of CD^**^			
Specialist in gastroenterology	17/108 (16%)	16%	
Serological assays	108/108 (100%)	100%	Recommended as the first tool to identify patients with symptoms and signs suggestive of CD for further diagnostic workup
In cases of positive serological test; referral to specialist in gastroenterology for final diagnosis.	96/108 (89%)	90%	If anti-TG2 antibody testing is positive, then patients should be referred to a pediatric gastroenterologist for further diagnostic workup
Final decision of intestinal biopsy by specialist in gastroenterology	106/108 (98%)	98%	
Treatment and follow-up**			
Recommend on gluten free diet only after diagnosis of CD	107/107 (100%)	100%	
Recommend yearly follow-up for physical growth	106/108 (98%)	98%	
Recommend follow-up by specialist in gastroenterology	69/108 (64%)	65%	

^*^Inverse probability weighting; ^**^Physicians who answered “always” or “usually”. *CD* Celiac disease, *IDA* Iron deficiency anemia, *IgA* Immunoglobulin A

## Discussion

The main findings of this survey are that (a) only 34 and 37% of the participants reported using the ESPGHAN/NASPGHAN guidelines on the diagnosis and treatment of *H. pylori* infection and the ESPGHAN guidelines on celiac disease, respectively, and (b) there was a high adherence to the guidelines for the management and treatment of celiac disease, but not of *H. pylori* infection.

Overall, the reported management of suspected celiac disease was consistent with the ESPGHAN guidelines [4]. All participants relied on serological assays for the initial diagnosis of celiac disease, and 89% would refer patients with positive celiac serology to a gastroenterology consultant for making the final diagnosis either by performing duodenal biopsies, or by immunoglobulin A TG2 antibody titers (> 10 times more than the upper normal limit), endomysial antibodies and human leukocyte antigen (HLA)-DQ2 and/or HLA-DQ8 [4].

On the other hand, testing for *H. pylori* infection in children with suspected duodenal ulcer, unexplained iron deficiency anemia and recurrent abdominal pain was reported by 78, 52 and 44% of the participants, respectively. Commonly, non-invasive testing (stool EIA and UBT) was the first choice for *H. pylori* infection, even though the guidelines recommend gastrointestinal endoscopy with biopsies for culture and histology [2, 3]. Similar to Chang et al. [6], we found that 79% of the participants recommended triple treatment for *H. pylori* infection, and 74% would prescribe it for 10–14 days, as currently recommended [3]. However, most participants prescribed clarithromycin, even though small-scale studies in Israeli children found high clarithromycin resistance in *H. pylori* isolates [7, 8]. This is inconsistent with the guideline to consider the local prevalence of antibiotic resistance of *H. pylori* strains.

In our study, 71% of the participants would refer to a consultant in gastroenterology incase of treatment failure. This finding might be consistent with the guideline [3] to individualize rescue therapy by considering antibiotic susceptibility, age of the child, and available antimicrobial options.

The main strengths of this study are its utilization of a questionnaire that was constructed by experts in epidemiology, survey methods and pediatric gastroenterology, and its being the first survey in Israel of the clinical practices primary-care pediatricians regarding the diagnosis and treatment of *H. pylori* infection and celiac disease. The main limitation of our study is its reliance on self-reported data that may not reflect actual practice. However, a reporting bias is unlikely to be different for the two illnesses assessed in the survey. A second limitation is the low response rate of the invited participants. Even though responders and non-responders were similar in demographic characteristics, we cannot rule out the possibility that they differed in practice habits.

## Conclusions

Future research should explore the causes of the limited adherence to practice guidelines for suspected *H. pylori* infection by Israeli primary-care pediatricians. Possible causes for this limited adherence are (a) Deficient understanding and implementation of the practice guidelines; this would require educational interventions. (b) Difficulties in implementing the practice guidelines in the Israeli primary care setting; this would require a review of the current guidelines with a view of their amendment. (c) Disagreement within the pediatric gastroenterology community; this would similarly require a review of the current guidelines for the management of suspected *H. pylori* infection with a view of forging a consensus on these guidelines.

## Supplementary information

**Additional file 1.** The questionnaire.

## Data Availability

Individual level data from this study cannot be made publically available due to legal and ethical restrictions.

## Abbreviations

EIA: Enzyme immunoassay
ESPGHAN: European Society for Pediatric Gastroenterology, Hepatology and Nutrition
*H. pylori*: *Helicobacter pylori*
HLA: Human leukocyte antigen
IDA: Iron deficiency anemia
IgA: Immunoglobulin A
MHS: Maccabi Healthcare Services
NASPGHAN: North American Society for Pediatric Gastroenterology, Hepatology and Nutrition
PPI: Proton pump inhibitors
SD: Standard deviation
UBT: Urea breath test
