# Supplementary material for: A survey of primary-care pediatricians regarding the management of Helicobacter pylori infection and celiac disease
Source: Isr J Health Policy Res. 2019 Dec 27;8:88. doi: 10.1186/s13584-019-0357-x (PMC6933930; doi:10.1186/s13584-019-0357-x)
Supplement: Supplementary file 1 — Additional file 1. The questionnaire. [file 13584_2019_357_MOESM1_ESM.docx]

**Appendix 1- Pediatricians questionnaire**

**Module I**: The following questions are related to the diagnosis and treatment of *Helicobacter pylori* infection in children. **Please, choose only one answer in all the following questions.**

**1. Do you use any guidelines for the diagnosis of *H. pylori* infection?**

1) No

2) Yes, please specify which guidelines: _____________________________________

**2.** **In the following situations, how often do you usually refer children to the diagnosis of *H. pylori*?**

|  | Always | Usually, yes | Usually, no | Never |
| --- | --- | --- | --- | --- |
| Suspected duodenal ulcer | 4 | 3 | 2 | 1 |
| First degree relatives of gastric cancer patients | 4 | 3 | 2 | 1 |
| Unexplained iron deficiency anemia | 4 | 3 | 2 | 1 |
| Recurrent abdominal pain  (Functional abdominal pain) | 4 | 3 | 2 | 1 |

**3**. **In children suspected to have *H. pylori* infection, the first test I usually choose is (please select one answer):**

1) Specialist in gastroenterology

2) Gastroscopy

3) Urea Breath Test

4) Stool antigen enzyme immunoassay

5) Serology

6) Other, please specify: ____________________

**4**. **What is the first-line treatment that you usually prescribe to a child with a positive gastroscopy for *H. pylori*, when you decide to treat?**

1) Triple therapy with PPIs / Clarithromycin / Amoxicillin

2) Triple therapy with PPIs / Amoxicillin / Metronidazole

3) Quadruple with PPIs / Clarithromycin / Amoxicillin / Metronidazole

4) Quadruple based on Bismuth

5) Refer the child to a specialist in gastroenterology

6) Other; please specify: ____________________

**5**. **For how many days do you prescribe the treatment?**

1) 7 days

2) 10 days

3) 14 days

4) Other; please specify: ____________________

**6**. **Do you usually refer the child to follow-up test after completing *H. pylori* treatment?**

1) Do not refer to additional tests if symptoms resolved

2) Refer to specialist in gastroenterology at least 1 month after therapy

3) Refer the child to urea breath test at least 1 month after therapy

4) Refer to perform stool antigen enzyme immunoassay at least 1 month after therapy

5. Other; please specify: ____________________

**7. What do you usually do in patients with positive *H. pylori* result on follow-up test?**

1) Do not refer to additional tests if symptoms resolved

2) Recommend the same treatment for a longer duration

3) Refer the child to specialist in gastroenterology

4) Recommend a different treatment; please specify: _____________________

5) Other; please specify: ____________________

**Module II**: The following questions are related to the diagnosis and treatment of celiac disease in children.

**1. Did you use any guidelines for the diagnosis and treatment of celiac?**

1) No

2) Yes, please specify: ______________________________________________

**2. Common symptoms that raise the suspicion of celiac are (you can choose more than one answer):**

1) Chronic/intermittent diarrhea

2) Growth impairment

3) Iron deficiency anemia

4) Abdominal pain

5) Acute bloody diarrhea

**3. In which of the following situations, celiac screening is recommended?**

1) Autoimmune diseases, e.g., type 1 diabetes

2) Down syndrome

3) First-degree relatives of celiac disease patients

4) First-degree relatives of gastric cancer patients

**4.** **How often do you choose each of the following in suspected cases of celiac disease for diagnosis and treatment of celiac? (Please choose only one answer):**

|  | Always | Usually, yes | Usually, no | Never |
| --- | --- | --- | --- | --- |
| Specialist in gastroenterology (first choice) | 4 | 3 | 2 | 1 |
| Serological assays (first choice) | 4 | 3 | 2 | 1 |
| In cases of positive serological test; referral to specialist in gastroenterology for final diagnosis. | 4 | 3 | 2 | 1 |
| Final decision of intestinal biopsy is made by specialist in gastroenterology | 4 | 3 | 2 | 1 |
| Recommend on gluten free diet before intestinal biopsy | 4 | 3 | 2 | 1 |
| Recommend on gluten free diet only after diagnosis of celiac disease | 4 | 3 | 2 | 1 |
| Recommend yearly follow-up for growth and disease complications | 4 | 3 | 2 | 1 |
| Recommend regular follow-up by specialist in gastroenterology | 4 | 3 | 2 | 1 |
